# Editorial: Opioids and opioid receptors in pain, addiction, and mood disorders

**DOI:** 10.3389/fpsyt.2024.1382894

**Published:** 2024-03-13

**Authors:** Yu Qiu, Yu-Jun Wang

**Affiliations:** ^1^ Department of Pharmacology and Chemical Biology, Shanghai Jiao Tong University School of Medicine, Shanghai, China; ^2^ Shanghai Institute of Materia Medica, Chinese Academy of Sciences, Shanghai, China; ^3^ Shandong Laboratory of Yantai Drug Discovery, Bohai Rim Advanced Research Institute for Drug Discovery, Yantai, China

**Keywords:** μ opioid receptor (MOR), addiction, obsessesive-compulsive disorder, oxycodone, tolerance

Clinically, opioids are used primarily for the management of moderate to severe pain because of their strong analgesic effects. At the same time, opioids are highly addictive, and opioid overprescription has caused serious health, social, and economic problems worldwide. Oxycodone is one such semi-synthetic opioid drug. It was widely used in North America in the 1990s as it was thought to have fewer side effects than morphine. However, its use was later found to pose serious health and addiction risks. Marie and Noble conducted a review showing that oxycodone has a lower affinity for the μ opioid receptor (MOR) than morphine. However, its metabolite oxymorphone has a higher affinity for MOR with greater efficacy and potency than oxycodone. Oxycodone also penetrates the blood-brain barrier faster and better than morphine. These factors may give it a greater potential for abuse.

Even though opioid abuse has drawn worldwide attention, opioid abuse, or opioid use disorder, is still the severe situation around the world. Rauschert et al. classified patients using prescribed opioid analgesics to identify those subjects that are prone to developing opioid use disorder. They found that patients with poor mental health or with a history of cannabis use had a higher prevalence of prescription opioid use disorder. Their findings will help prescribe opioids more carefully. On the other hand, for those with opioid use disorder, methadone is the most frequently used agonist to treat the condition. To better monitor treatment, Liu et al. constructed a model that integrates urine opiate levels, treatment adherence, and the likelihood of treatment discontinuation to optimize methadone dosing. The model allows for remote adjustment of methadone dosing, such as during the COVID-19 pandemic. Meanwhile, Li et al. studied the drug-related and negative-affective-related attentional bias of individuals under methadone maintenance therapy. They observed the normalized attentional bias of these patients, indicating that methadone maintenance therapy can ameliorate the abnormal attentional bias in patients with opioid use disorder. Moreover, methadone maintenance therapy also modulated the attentional avoidance of negative-affective cues and the level of impulsivity in the patients. These results supported the idea that methadone maintenance reduces relapses to drug abuse.

In addition, opioids regulate many other physiological functions in the central nervous system (CNS), including nociception, mood control, learning and memory, and so on. The high expression of endogenous opioid peptides and receptors in the limbic system highlights the central role of the opioid system in both euphoric and reward processes, and emotional responses, making it a plausible target for intervention in mood disorders, especially anxiety and depression. In our Research Topic, one of the papers investigated the use of MOR agonists in obsessive–compulsive disorder (OCD). OCD is characterized by intrusive and disturbing thoughts (obsessions) and repetitive behaviors (compulsions) (1). The first-line treatment for OCD are serotonin reuptake inhibitors. MOR agonists can be used to treat patients who are resistant to serotonin reuptake inhibitors. Youngblood et al. studied the effect of EPD1504, a newly developed partial agonist of MORs, on OCD. EPD1504 alleviated OCD-like behaviors in two different rat models at a dose that occupies approximately 20% of MORs in the CNS. Moreover, effective doses of EPD1504 did not impair locomotor activity or respiration with less dependence. Buprenorphine has been tested in clinical trials for OCD and has shown promising treatment potential (2). However, buprenorphine has been found to cause respiratory depression and its use was restricted due to abuse liability (3). Thus, EPD1504 may be a better candidate for OCD treatment. Another drug, tianeptine, which enhances serotonin uptake, modulates glutamate neurotransmission, and is effective in the treatment of depression and anxiety, has been proven to activate MOR (4). The work of Allain et al. showed that the analgesic, locomotor, and rewarding effects of tianeptine are mediated by MOR. Like other opioids, chronic administration of tianeptine could induce tolerance to its analgesic and hyperlocomotoric effects. These data provide a detailed description of tianeptine as a MOR agonist.

In conclusion, the current Research Topic covers opioid abuse and its treatment, in addition to the profiles of two relatively new MOR agonists. The works included here will help improve the clinical application of opioids and related drugs.

## Author contributions

YQ: Writing – original draft. Y-JW: Writing – review & editing.
